# ﻿Two new species of *Hyalonema* (Hexactinellida, Amphidiscosida, Hyalonematidae) from the Indo-West Pacific

**DOI:** 10.3897/zookeys.1262.164821

**Published:** 2025-12-03

**Authors:** Lin Gong, Edwin Setiawan, Xinzheng Li, Swee Cheng Lim

**Affiliations:** 1 Institute of Oceanology, Chinese Academy of Sciences, Qingdao 266071, China Institute of Oceanology, Chinese Academy of Sciences Qingdao China; 2 College of Marine Science, University of Chinese Academy of Sciences, Beijing, China University of Chinese Academy of Sciences Beijing China; 3 Laboratory of Animal Bioscience and Technology, Department of Biology, Institut Teknologi Sepuluh Nopember, Surabaya, Indonesia Institut Teknologi Sepuluh Nopember Surabaya Indonesia; 4 Laboratory for Marine Biology and Biotechnology, Qingdao National Laboratory for Marine Science and Technology, Qingdao, China Qingdao National Laboratory for Marine Science and Technology Qingdao China; 5 Lee Kong Chian Natural History Museum, National University of Singapore, 2 Conservatory Drive, Singapore 117377, Singapore National University of Singapore Singapore Singapore

**Keywords:** Deep sea, glass sponge, Hyalonema (Cyliconema) dexiangi sp. nov., Hyalonema (Cyliconema) subconica sp. nov., new species, phylogeny, taxonomy

## Abstract

This study describes two new species collected from deep-sea environments in the Indo-West Pacific. Hyalonema (Cyliconema) dexiangi**sp. nov.**, collected from the Indian Ocean, is characterized by distinctive finger-like protuberances on the body surface, the presence of choanosomal pentactins, the co-occurrence of micropentactins and microhexactins, and the absence of mesamphidiscs. Hyalonema (Cyliconema) subconica**sp. nov.**, sampled from a seamount in the western Pacific Ocean, is distinguished by the presence of pinular diactins, a unique combination of choanosomal spicules (diactins, pentactins, and hexactins), and the presence of basalia bearing two-toothed anchors. In addition to their morphological distinctions, molecular phylogenetic analyses based on 16S rDNA sequences indicate a close genetic relationship between the two species.

## ﻿Introduction

*Hyalonema* Gray, 1832 is the most speciose genus within the class Hexactinellida, comprising 13 subgenera and 119 species (De Voogd et al. 2025). More than 90 species were described prior to 1930, based primarily on material collected during historical expeditions. These include the HMS Challenger global survey (1873–1876) (Schulze 1886, 1887), the Valdivia Expedition (1898–1899) off West Sumatra (Schulze 1904), and the Siboga Expedition (1899–1900) in the Banda Sea region (Ijima 1927). Distinct morphological characteristics differentiate some subgenera of *Hyalonema* (e.g., H. (Prionema) Lendenfeld, 1915; H. (Paradisconema) Ijima, 1927; and H. (Oonema) Lendenfeld, 1915), while diagnostic features remain ambiguous in certain subgenera, notably H. (Coscinonema) Ijima, 1927. Taxonomic revision of *Hyalonema* presents significant challenges due to its remarkable species richness and the historical status of many type specimens. Molecular studies on *Hyalonema* remain limited. To date, only 16S rDNA sequences from representatives of five subgenera (H. (Corynonema), H. (Prionema), H. (Cyliconemaoida), H. (Cyliconema), H. (Onconema)) have been analysed, with few molecular data available for species-level identification (Kersken et al. 2018a; Dohrmann 2019).

**Figure 1.**
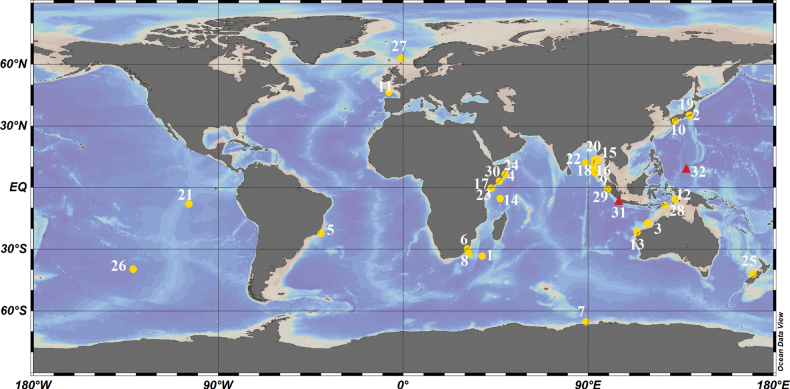
Type locations of species of Hyalonema (Cyliconema): 1. H. (Cyliconema) abyssale; 2. H. (Cyliconema) apertum; 3. H. (Cyliconema) clavapinulatum; 4. H. (Cyliconema) coniforme; 5. H. (Cyliconema) conqueror; 6. H. (Cyliconema) curvisclera; 7. H. (Cyliconema) drygalskii; 8. H. (Cyliconema) eupinnulum; 9. H. (Cyliconema) globiferum; 10. H. (Cyliconema) hozawai; 11. H. (Cyliconema) infundibulum; 12. H. (Cyliconema) keiense; 13. H. (Cyliconema) lanceolatum; 14. H. (Cyliconema) madagascarense; 15. H. (Cyliconema) martabanense; 16. H. (Cyliconema) masoni; 17. H. (Cyliconema) molle; 18. H. (Cyliconema) nicobaricum; 19. H. (Cyliconema) ovatum; 20. H. (Cyliconema) pirum; 21. H. (Cyliconema) polycaulum; 22. H. (Cyliconema) rapa; 23. H. (Cyliconema) simile; 24. H. (Cyliconema) somalicum; 25. H. (Cyliconema) tasmani; 26. H. (Cyliconema) tenerum; 27. H. (Cyliconema) thomsonis; 28. H. (Cyliconema) timorense; 29. H. (Cyliconema) tulipa; 30. H. (Cyliconema) valdiviae; 31. H. (Cyliconema) dexiangi sp. nov.; 32. H. (Cyliconema) subconica sp. nov.

Hyalonema (Cyliconema) Ijima, 1927 is characterized by dermal spicules bearing whip-like pinular rays, macramphidiscs with umbels broader than long, and the absence of both ambuncinates and a sieve-plate (Tabachnick and Menshenina 2002). The subgenus comprises 30 species with a broad geographical distribution (Fig. 1), based on the locations of their holotypes, spanning the Pacific, Atlantic, and Indian Oceans (Suppl. material 1). Notably, most species occur in the Indian Ocean, with over half recorded at depths exceeding 1000 m. The World Porifera Database (https://www.marinespecies.org/porifera/) lists relatively few species from the western Pacific Ocean. Six valid H. (Cyliconema) species were recorded from Indonesian waters: H. (Cyliconema) apertum Schulze, 1886 from the Banda Sea and West Sumatra; H. (Cyliconema) keiense Ijima, 1927, and H. (Cyliconema) timorense Ijima, 1927 collected during the Siboga Expedition in the Banda Sea; H. (Cyliconema) martabanense Schulze, 1900, H. (Cyliconema) rapa Schulze, 1900, and H. (Cyliconema) tulipa Schulze, 1904, all obtained during the Valdivia Expedition off West Sumatra.

Species of *Hyalonema* predominantly inhabit muddy benthic environments across the world’s oceans (Tabachnick and Menshenina 2024) and have been reported in polymetallic nodule fields (Kersken et al. 2018b; Stratmann et al. 2021). In 2013, a research cruise conducted by the Institute of Oceanology, Chinese Academy of Sciences, to the Yap Seamount in the northwestern Pacific Ocean collected a single specimen of Hyalonema (Cyliconema) using the remotely operated vehicle (ROV) Fa Xian. A second specimen was obtained during the South Java Sea Expedition (SJADES) in 2018, jointly organized by the Lee Kong Chian Natural History Museum (LKCNHM) at the National University of Singapore (NUS) and the Indonesian Research Agency (BRIN). Integrated morphological and molecular analyses confirm that these specimens represent two new species, which are formally described and illustrated here. We also provide the corresponding 16S rDNA and COI sequences. However, given the current scarcity of molecular data for *Hyalonema*, establishing a comprehensive phylogenetic framework for the genus remains difficult.

## ﻿Material and methods

One sponge sample was collected in the Indian Ocean using a beam trawl during the SJADES expedition from 23 March to 5 April 2018 (Chim et al. 2021; Ng and Rahayu 2021). The sample was deposited at the Lee Kong Chian Natural History Museum (**LKCNHM**), Singapore. One sponge sample was collected by the submersible ROV Fa Xian during a cruise of the research ship Ke Xue in the western Pacific Ocean. The sample was deposited in the Marine Biological Museum of the Chinese Academy of Sciences (**MBM**) at the Institute of Oceanology of the Chinese Academy of Sciences (**IOCAS**).

Total genomic DNA was extracted using the Tissue DNA Kit (OMEGA Bio-Tek) according to manufacturer’s protocols. The libraries were finally sequenced using a next-generation sequencing platform (Illumina) with PE150 mode according to the standard protocols. Raw sequence reads were quality-trimmed using Trimmomatic (Bolger et al. 2014) to remove adaptor contaminants and low-quality reads. The clean reads were then assembled into a set of contigs for each sample using MegaHit v. 1.2.9 (Li et al. 2015). Finally, the 16S rDNA and COI genes were recovered from the assembled contigs.

A phylogenetic tree was constructed from partial 16S rDNA sequences of *Hyalonema*. The reference sequence alignment for the 16S rDNA gene was obtained from the GitHub repository by Dohrmann et al. (2023) (https://github.com/PalMuc/SONNE_Hexactinellida/blob/main/16S.fasta). This dataset was subsequently curated to exclude all representatives of the subclass Hexasterophora, with the pheronematid *Semperella jialongae* retained as the outgroup for rooting the tree. The untrimmed and trimmed alignments are provided in Suppl. materials 2, 3. We used the workflow desktop platform PhyloSuite (Zhang et al. 2020) to build the phylogenetic trees. For Bayesian inference (BI) and maximum-likelihood (ML) analyses, the best-fit substitution models were HKY+G and TIM2+R inferred by ModelFinder (Kalyaanamoorthy et al. 2017). For the ML analysis, we employed IQ-TREE v. 1.6.8 (Nguyen et al. 2015), with node support assessed through ultrafast bootstrap approximation (UFBoot) based on 1000 replicates. The BI tree was reconstructed using MrBayes v. 3.2 (Ronquist et al. 2012). The analysis consisted of two independent runs of four Markov chains each, each running for 10,000,000 iterations, with sampling every 1000 iterations. After discarding the first 25% of trees as burn-in, the remaining trees were used to construct the 50% majority-rule consensus tree and to estimate posterior probabilities (PPs). Phylogenetic tree annotation was performed using the webtool iTOL (https://itol.embl.de/).

## ﻿Results

### ﻿Taxonomy

**Family Hyalonematidae Gray, 1857**

**Genus *Hyalonema* Gray, 1832**

**Subgenus Hyalonema (Cyliconema) Ijima, 1927**

#### ﻿ Hyalonema (Cyliconema) dexiangi

Taxon classificationAnimaliaAmphidiscosidaHyalonematidae

Gong, Setiawan & Lim sp. nov.

F36C9AD5-3966-5753-A366-51F4C7C8A95B

https://zoobank.org/0EAE2FA7-771B-4698-AEB2-14EA1ADBBAC8

Figs 2, 3, Table 1

##### Material examined.

***Holotype***: ZRC.POR.0519. Station CP23 (6°46.739'S, 105°09.239'E). sample number CP2301. 30 March 2018, collected by Lim Swee-Cheng. Beam Trawl. Depth 571 m, gravel and mud substrates. Indian Ocean, south of Cilacap (West Java, Indonesia).

##### Description.

**Figure 2.**
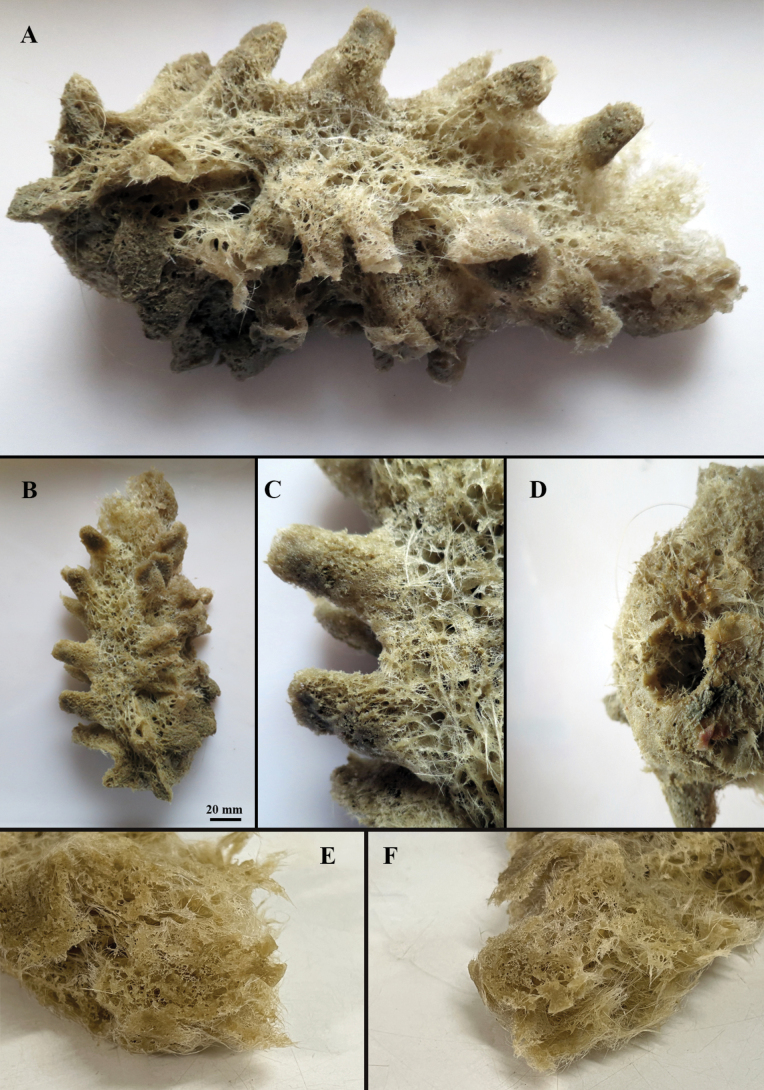
Hyalonema (Cyliconema) dexiangi sp. nov. A, B. External morphology of the specimen; C. Finger-like protuberances of the specimen; D. Holes left after the basalia torn off during bottom trawling; E, F. Atrial surface.

The sponge exhibits a cylindrical form (Fig. 2A, B) with finger-like protuberances on its surface (Fig. 2C), each terminating in an osculum (diameter 12 mm). The apex of the specimen exhibits a finger-like form without a terminal osculum or sieve-plate (Fig. 2E, F). Due to trawling collection, the specimen is morphologically damaged and lacks basalia (Fig. 2D). The specimen measures 224 mm in body length and 106 mm in width. The dermal surface displays relatively uniform mesh openings, ranging from 3 mm to 6 mm in diameter.

**Table 1.** Measurements of the spicules of Hyalonema (Cyliconema) dexiangi sp. nov. (in µm); *N* = number of spicules measured; s.d. = standard deviation; range = range from the minimum to the maximum.

	*N*	mean	range	s.d.
Dermalia, pinule				
pinular ray length	20	248	117–384	89
pinular ray width	20	8	5–11	2
tangential ray length	20	32	21–40	5
tangential ray width	20	6	4–10	1
Atrialia, pinule				
pinular ray length	20	215	170–282	32
pinular ray width	20	7	6–8	0.7
tangential ray length	20	27	17–41	6
tangential ray width	20	5	4–7	0.7
Canalaria, pinule				
pinular ray length	20	219	96–367	79
pinular ray width	20	7	4–10	2
tangential ray length	20	34	23–52	8
tangential ray width	20	6	3–7	1
Hypodermalia, pentactin				
tangential ray length	20	349	257–442	47
tangential ray width	20	18	14–21	2
Hypoatrialia, pentactin				
tangential ray length	8	294	224–336	35
tangential ray width	8	15	12–17	2
Choanosomalia, diactin				
length	20	1812	1140–2883	512
width	20	13	9–18	13
Choanosomalia, pentactin				
tangential ray length	15	384	276–600	93
tangential ray width	15	21	15–44	7
Pinular diactin				
length	3	824	712–1029	178
width	3	8	7–8	0.4
Macramphidisc				
length	12	329	228–380	362
umbel length	12	95	80–106	8
umbel diameter	12	131	110–145	10
Micramphidisc				
length	20	17	14–21	2
umbel length	20	6	4–8	1
umbel diameter	20	6	5–8	0.7
Microhexactin				
length	20	62	41–98	13
Micropentactin				
length	20	61	27–86	15

##### Spicules.

**Figure 3.**
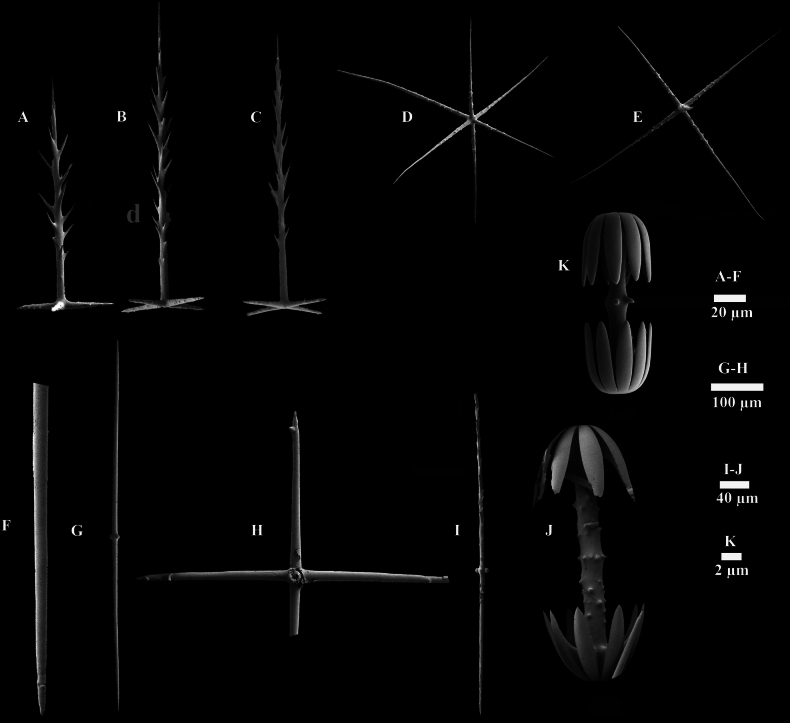
SEM images of spicules of Hyalonema (Cyliconema) dexiangi sp. nov. A. Atrial pinular pentactin; B. Dermal pinular pentactin; C. Canalarial pinular pentactin; D. Microhexactin; E. Micropentactin; F. Details of diactin; G. Diactin; H. Dermal pentactin; I. Pinular diactin; J. Macramphidisc; K. Micramphidisc.

Atrialia are pinular pentactins (Fig. 3A) with longer spines on the pinular rays (170–282 μm in length) and tangential rays (17–41 μm in length) bearing small spines. Dermalia are pinular pentactins (Fig. 3B), elongated and whip-like, featuring pinular rays (117–384 μm in length) with longer spines and tangential rays (21–40 μm in length) bearing small spines. Canalaria are pinular pentactins (Fig. 3C) with spines on the pinular rays (96–367 μm in length), and tangential rays (23–52 μm in length) bearing small spines. The choanosomal skeleton consists of diactins and pentactins. The diactins (Fig. 3G) have smooth surfaces, pointed ends (Fig. 3F), and some exhibit a medial swelling (1140–2883 µm in length). Hypodermalia (Fig. 3H) and hypoatrialia are pentactins with smooth rays, and hypodermalia pentactins (257–442 μm in length) are more abundant than hypoatrialia pentactins (224–336 μm in length). Choanosomal pentactins (288–575 μm in length) with smooth rays are occasionally present, and hexactins are rarely observed. Pinular diactins, which are extremely scarce, have a pinular ray densely covered with spines (Fig. 3I), with the other end pointed and medially tuberculate, measuring 712–1029 μm in length.

Microscleres are two types of amphidiscs, micropentactins and microhexactins. Macramphidiscs (Fig. 3J) have smooth disc surface and tuberculated axial surface, measuring 228–380 μm in length. The disc diameter (110–145 μm) is greater than the disc length (80–106 μm). Micramphidiscs (Fig. 3K) (14–21 μm in length) possess palmate heads and nearly smooth shafts sparsely covered with spines. Micropentactins (Fig. 3E) have four tangential rays (41–98 μm in length) of approximately equal length. Microhexactins (Fig. 3D) have rays (27–86 μm in length) with slightly curved tips.

##### Etymology.

Dexiangi is named in honor of the late Professor Wang Dexiang from Xiamen University. A passionate and dedicated sponge researcher, Professor Wang made significant contributions to sponge science and mentored numerous students in China. Remembered for his generosity, kindness, and collegial spirit, his passing represents a profound loss to the scientific community and all who knew him.

##### Type locality.

Indian Ocean south of Java, 571 m.

##### GenBank number.

PV625033 (16S rDNA) and PV618251 (COI).

##### Molecular data.

**Figure 4.**
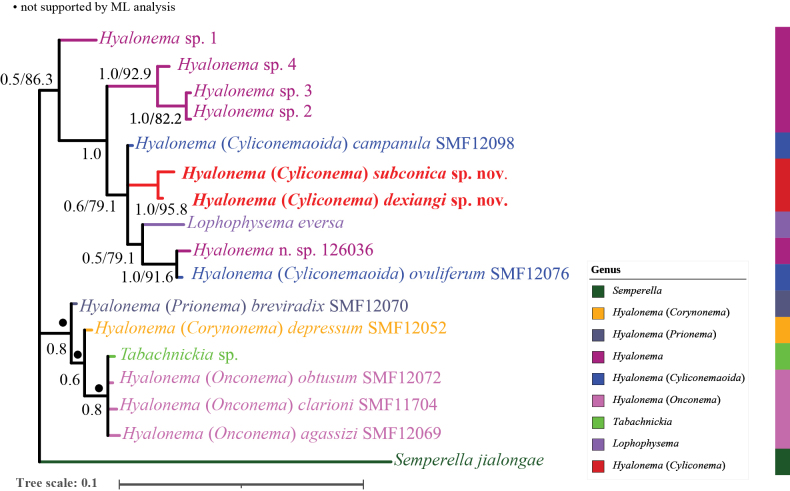
Phylogenetic tree obtained by Bayesian inference (BI) analysis based on 16S rDNA. The numbers at each node are Bayesian posterior probabilities (PP, left) and UFBoot (right) values. Support values under 50% are not shown.

This study obtained molecular sequences of two new species of H. (Cyliconema). In the Bayesian inference (BI) tree based on 16S rDNA sequences (Fig. 4), the new species groups with another new species described below. Currently available *Hyalonema* sequences in GenBank represent five subgenera. Phylogenetic analysis revealed that H. (Cyliconema) groups with H. (Cyliconemaoida), along with five unidentified *Hyalonema* species and *Lophophysema eversa* Gong, Li & Qiu, 2014. The topology of the maximum-likelihood (ML) tree was largely congruent with that of the BI tree, as shown in Suppl. material 4.

##### Remarks.

The new species exhibits dermal spicules with whip-like pinular rays, macramphidiscs displaying umbels broader than long, and an absence of ambuncinates and sieve-plate, which align with the diagnostic characteristics of Hyalonema (Cyliconema). Within the subgenus Hyalonema (Cyliconema), only three species – H. (Cyliconema) ovatum Ijima, 1895, H. (Cyliconema) tulipa Schulze, 1904 and H. (Cyliconema) valdiviae Schulze, 1904 – lack mesamphidiscs, a trait consistent with the new species. However, the new species possesses micropentactins, which have not been reported in these three species. Notably, H. (Cyliconema) tulipa, also recorded in Indonesian waters, exhibits strongly curved rays of microhexactins and features a vertically protruding central cone on its atrial surface. In contrast, the microhexactins of the new species show only slight curvature at the ray tips and lack a central cone on the atrial surface. Externally, the sponge exhibits finger-like protuberances on its surface, a morphology closely resembling that of *Composocalyx* Schulze, 1904, which distinguishes it from species within the genus *Hyalonema*. The new species is further characterized by the presence of choanosomal pentactins, a feature reported in only a few species, such as H. (Onconema) clarioni Kersken, Janussen & Martínez Arbizu, 2018 and H. (Prionema) breviradix Kersken, Janussen & Martínez Arbizu, 2018, and this distinguishes it readily from other *Hyalonema* species. These morphological differences confirm the status of this specimen as a new species.

#### ﻿ Hyalonema (Cyliconema) subconica

Taxon classificationAnimaliaAmphidiscosidaHyalonematidae

Gong, Li & Lim sp. nov.

A3B17497-2063-561F-AF10-76297BDA7C5A

https://zoobank.org/165AFAC1-9F1A-46F3-B472-6B937C859E8D

**Figure 5.**
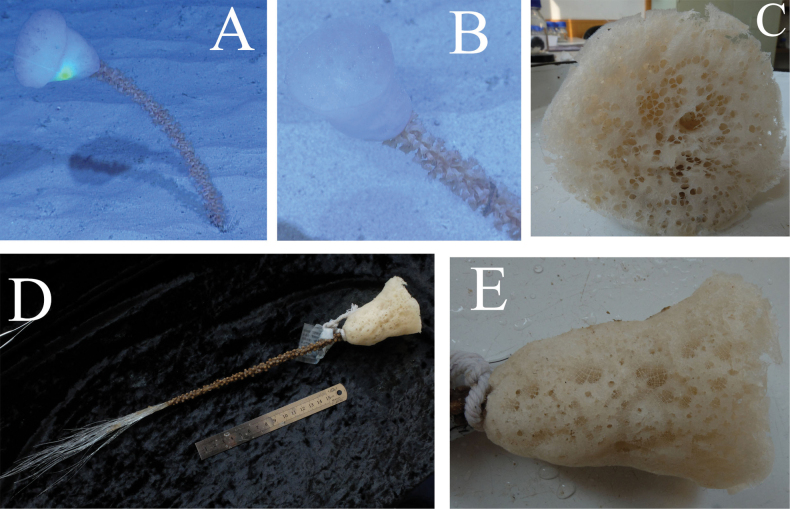
Hyalonema (Cyliconema) subconica sp. nov. A, B. Photograph showing its natural growth form on the seabed; C–E. External morphology of the specimen after being lifted out of seawater.

**Figure 6.**
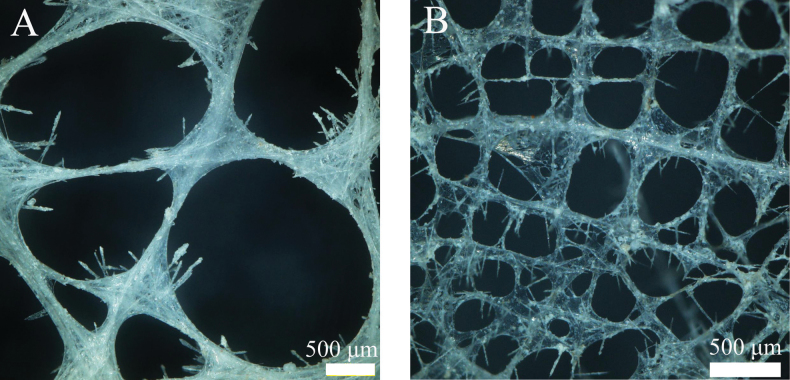
The skeleton of Hyalonema (Cyliconema) subconica sp. nov. A. Atrial skeleton; B. Dermal skeleton.

**Figure 7.**
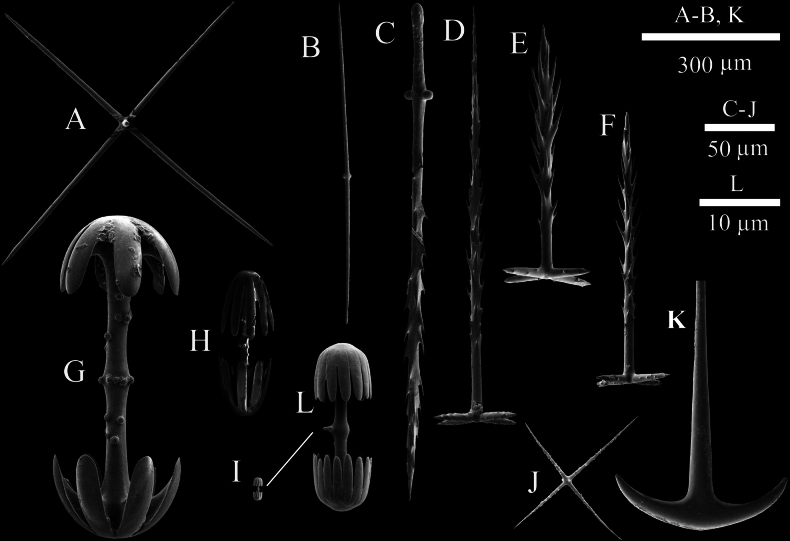
SEM images of spicules of Hyalonema (Cyliconema) subconica sp. nov. A. Choanosomal pentactin; B. Diactin; C. Pinular diactin; D. Dermal pinular pentactin; E. Atrial pinular pentactin; F. Canalarial pinular pentactin; G. Macramphidisc; H. Mesamphidisc; I. Micramphidisc; J. Micropentactin; K. Basalia; L. Details of the micramphidisc.

Figs 5, 6, 7, Table 2

##### Material examined.

***Holotype***: MBM288250. 15 December 2014, Depth 1106 m, foraminiferal ooze substrate. Yap seamount (8°51.66'N, 137°44.09'E).

##### Description.

The sponge is white, anchored to the seafloor of foraminiferal ooze by a cluster of intertwined basalia (Fig. 5A, B). The sponge is bell-shaped, resembling a rose (Fig. 5C). The surface of the basalia is colonized by anemones (Fig. 5D). The basalia measure nearly 39 cm in length, while the main body of the sponge is 8 cm long, with an atrial surface diameter of 6 cm. The atrial surface has relatively uniform mesh openings (Fig. 6A), with pore diameters ranging from 0.3 mm to 2.3 mm. The mesh openings are separated by non-porous tissue of uneven distribution, varying in width from 0.5 mm to 5 mm. The dermal areas display distinct mesh patterns (Figs 5E, 6B) that are smaller than those of the sieve-plates, with pore diameters 0.1 mm to 0.6 mm.

**Table 2.** Measurements of the spicules of Hyalonema (Cyliconema) subconica sp. nov. (in µm); *N* = number of spicules measured; s.d. = standard deviation; range = range from the minimum to the maximum.

	*N*	mean	range	s. d.
Dermalia, pinule				
pinular ray length	30	235	206–277	17
pinular ray width	30	6	5–7	1
tangential ray length	30	23	18–29	3
tangential ray width	30	5	4–5	0.4
Atrialia, pinule				
pinular ray length	25	197	147–252	24
pinular ray width	25	8	5.5–9.5	1
tangential ray length	25	30	23–40	3
tangential ray width	25	5	4–6	0.6
Canalaria, pinule				
pinular ray length	20	211	186–240	16
pinular ray width	20	5	4–6	0.6
tangential ray length	20	27	22–36	4
tangential ray width	20	4	3–6	0.5
Hypodermalia, pentactin				
tangential ray length	16	403	288–575	87
tangential ray width	16	16	12–23	3
Hypoatrialia, pentactin				
tangential ray length	6	381	266–624	141
tangential ray width	6	16	11–19	3
Choanosomalia, diactin				
tangential ray length	30	1387	893–1977	259
tangential ray width	30	9	6–11	1
Choanosomalia, pentactin				
length	12	461	225–789	196
width	12	13	9–16	2
Choanosomalia, hexactin				
length	6	320	270–377	40
Pinular diactin				
length	30	492	273–708	108
width	30	9	7–12	1
Macramphidisc				
length	7	229	220–244	7
umbel length	7	66	56–73	5
umbel diameter	7	93	80–101	7
Mesamphidisc				
length	22	57	39–69	8
umbel length	22	19	13–27	3
umbel diameter	22	15	11–19	2
Micramphidisc				
length	25	16	13–21	2
umbel length	25	5	4–7	0.9
umbel diameter	25	6	4–7	0.7
Micropetactin				
ray length	5	53	37–76	15

##### Spicules.

The choanosomal skeleton primarily consists of diactins (Fig. 7B) (893–1977 µm in length), which have smooth surfaces, pointed ends, and occasionally exhibit medial swellings. Pentactins (ray length: 288–575 μm) and hexactins (ray length: 270–377 μm) with smooth rays are occasionally observed. Dermalia are pinular pentactins (Fig. 7D), elongated and whip-like, featuring pinular rays (206–277 μm in length) with short spines and tangential rays (18–29 μm in length) bearing small spines. Atrialia are pinular pentactins (Fig. 7E) with longer spines on the pinular rays (147–252 μm in length) and tangential rays (23–40 μm in length) bearing small spines. Hypodermalia (288–575 μm in length) and hypoatrialia (266–624 μm in length) are pentactins with smooth rays, like choanosomal pentactins (Fig. 7A). Canalaria are pinular pentactins (Fig. 7F) with spines on the pinular rays (186–240 μm), and tangential rays (22–36 μm in length) bearing small spines. Pinular diactins (273–708 μm in length) have a pinular ray densely covered with spines (Fig. 7C), with the other end rounded or pointed, medially tuberculate. The basalia were predominantly broken during collection, making it impossible to observe the terminal structures. And, on rare occasions, one or two slender spicules were observed to display two-toothed anchors (Fig. 7K).

Microscleres are three types of amphidiscs and micropentactins. Macramphidiscs (Fig. 7G) are oval-shaped, measuring 220–244 μm in length, with smooth disc surfaces and tuberculated axial surface. The disc diameter (80–101 μm) is greater than the disc length (56–73 μm). Mesamphidiscs (Fig. 7H) measure 39–69 μm in length, with more teeth on the terminal discs compared to macramphidiscs. The axial surface exhibits numerous tubercles, and the disc diameter (11–19 μm) is smaller than the disc length (13–27 μm). Micramphidiscs (Fig. 7I) (13–21 μm in length) have disc diameters (4–(5)–7 μm) smaller than disc lengths (4–(6)–7 μm), and the teeth are densely arranged. Micropentactins (Fig. 7F), which are extremely scarce, possess four tangential rays (37–76 μm in length) of approximately equal length. We observed only one microhexactin among spicules from different parts of the sponge sample, suggesting that microhexactins are nearly absent in this species.

##### Etymology.

The new species is named after its external morphology. *Subconica* refers to the subconical external morphology of this new species.

##### Type locality.

Yap Seamounts (western Pacific) with foraminiferal ooze substrate, 1106 m.

##### GenBank number.

PV625035 (16S rDNA) and PV618253 (COI).

##### Molecular data.

In the BI tree (Fig. 4) based on the 16S rDNA sequences, the new species grouped with H. (Cyliconema) dexiangi sp. nov., demonstrating their close phylogenetic relationship.

##### Remarks.

During the identification of this new species, it was observed that its dermal pinular rays are thickest at the base and taper distally, while the macramphidiscs possess umbels that are broader than long. These characteristics align well with the diagnostic features of the subgenera H. (Cyliconema) and H. (Coscinonema). When Ijima established these two subgenera in 1927, he noted that H. (Coscinonema) possesses a sieve-like covering layer with uniformly distributed small open meshes in all species, and contains ambuncinates, whereas H. (Cyliconema) lacks an oscular sieve-plate or atrial covering layer and does not possess ambuncinates. However, among the 13 species under H. (Coscinonema), only one species has been reported to contain ambuncinates. Conversely, among the 30 species within H. (Cyliconema), several species (including H. (Cyliconema) coniforme Schulze, 1904, H. (Cyliconema) conqueror Tabachnick, Menshenina, Lopes & Hajdu, 2009, H. (Cyliconema) globiferum Schulze, 1904, and H. (Cyliconema) simile Schulze, 1904) have been found to possess sieve-plates. Therefore, it seems that there are no distinct morphological characteristics that reliably differentiate H. (Coscinonema) and H. (Cyliconema). Although the new species exhibits a sieve-plate, we assign it to H. (Cyliconema) based on the absence of ambuncinates. Molecular data further indicate a close phylogenetic relationship between this new species and H. (Cyliconema) dexiangi sp. nov., supporting its classification within the subgenus H. (Cyliconema), despite the presence of a sieve-plate in the new species.

Hyalonema (Cyliconema) subconica sp. nov. possesses unique diagnostic features that easily distinguish it from other species within the subgenera H. (Cyliconema) and H. (Coscinonema): 1) it contains pinular diactins with rounded tips (occasionally observed), whereas other species exclusively exhibit pointed tips; 2) the quantity of micropentactins is extremely low and microhexactins are nearly absent, while all other species in the subgenus possess microhexactins; and 3) the presence of choanosomal pentactins easily distinguishes it from its congeners. The new species exhibits a conical form, with an external morphology most closely resembling the holotype of H. (Cyliconema) conqueror, however, they differ in spicule composition. Specifically, the new species possesses micropentactins and virtually lacks microhexactins, whereas H. (Cyliconema) conqueror contains microhexactins, micropentactins, and microstauractins.

## ﻿Conclusion

In this study, we describe two new species of Hyalonema (Cyliconema) and provide complete COI and 16S rDNA sequences for each specimen. Due to the limited availability of molecular data for most *Hyalonema* species – many represented in GenBank solely by 16S rDNA sequences and just four COI sequences from unidentified species in Dohrmann et al. (2012) and Dohrmann et al. (2023) – we constructed a phylogenetic tree based only on the 16S rDNA marker. The resulting tree supported the non-monophyly of *Hyalonema*, consistent with the findings of Dohrmann (2019).

The lack of comprehensive molecular data, combined with the fact that many specimens were collected decades ago, continues to hinder a full taxonomic revision of the genus. Although COI amplification in Hexactinellida is generally difficult (Dohrmann et al. 2012), we advocate its broader application to amphidiscophoran taxa in future studies. Likewise, phylogenetic relationships within Amphidiscophora should be further investigated by incorporating more taxa of Hyalonematidae, Pheronematidae, and Monorhaphididae (Wörheide et al. 2012).

## Supplementary Material

XML Treatment for Hyalonema (Cyliconema) dexiangi

XML Treatment for Hyalonema (Cyliconema) subconica

## ﻿Additional information

### Use of AI

DeepSeek was employed in this paper for English language enhancement.

### Author ORCIDs

Lin Gong https://orcid.org/0000-0002-0003-3887

Edwin Setiawan https://orcid.org/0000-0003-3947-3334

Xinzheng Li https://orcid.org/0000-0001-6344-6542

Swee Cheng Lim https://orcid.org/0009-0008-4979-1348

### Data availability

All of the data that support the findings of this study are available in the main text or Supplementary Information.

## Supplementary materials

Supplementary material 1 Type locations of species of Hyalonema (Cyliconema) Data type xlsx https://binary.pensoft.net/file/1483738This dataset is made available under the Open Database License (http://opendatacommons.org/licenses/odbl/1.0/). The Open Database License (ODbL) is a license agreement intended to allow users to freely share, modify, and use this Dataset while maintaining this same freedom for others, provided that the original source and author(s) are credited.Lin Gong, Edwin Setiawan, Xinzheng Li, Swee Cheng Lim

Supplementary material 2 untrimmed alignments of 16S rDNA sequences of *Hyalonema* Explanation note fas https://binary.pensoft.net/file/1483739This dataset is made available under the Open Database License (http://opendatacommons.org/licenses/odbl/1.0/). The Open Database License (ODbL) is a license agreement intended to allow users to freely share, modify, and use this Dataset while maintaining this same freedom for others, provided that the original source and author(s) are credited.Lin Gong, Edwin Setiawan, Xinzheng Li, Swee Cheng Lim

Supplementary material 3 trimmed alignments of 16S rDNA sequences of *Hyalonema* Explanation note fas https://binary.pensoft.net/file/1483740This dataset is made available under the Open Database License (http://opendatacommons.org/licenses/odbl/1.0/). The Open Database License (ODbL) is a license agreement intended to allow users to freely share, modify, and use this Dataset while maintaining this same freedom for others, provided that the original source and author(s) are credited.Lin Gong, Edwin Setiawan, Xinzheng Li, Swee Cheng Lim

Supplementary material 4 Phylogenetic tree obtained by maximum-likelihood (ML) analysis based on 16S rDNA Data type tif https://binary.pensoft.net/file/1483741This dataset is made available under the Open Database License (http://opendatacommons.org/licenses/odbl/1.0/). The Open Database License (ODbL) is a license agreement intended to allow users to freely share, modify, and use this Dataset while maintaining this same freedom for others, provided that the original source and author(s) are credited.Lin Gong, Edwin Setiawan, Xinzheng Li, Swee Cheng Lim
