# Supplementary figures and images for: ﻿Two new species of Hyalonema (Hexactinellida, Amphidiscosida, Hyalonematidae) from the Indo-West Pacific
Source: Zookeys. 2025 Dec 3;1262:81–96. doi: 10.3897/zookeys.1262.164821 (PMC12696471; doi:10.3897/zookeys.1262.164821)

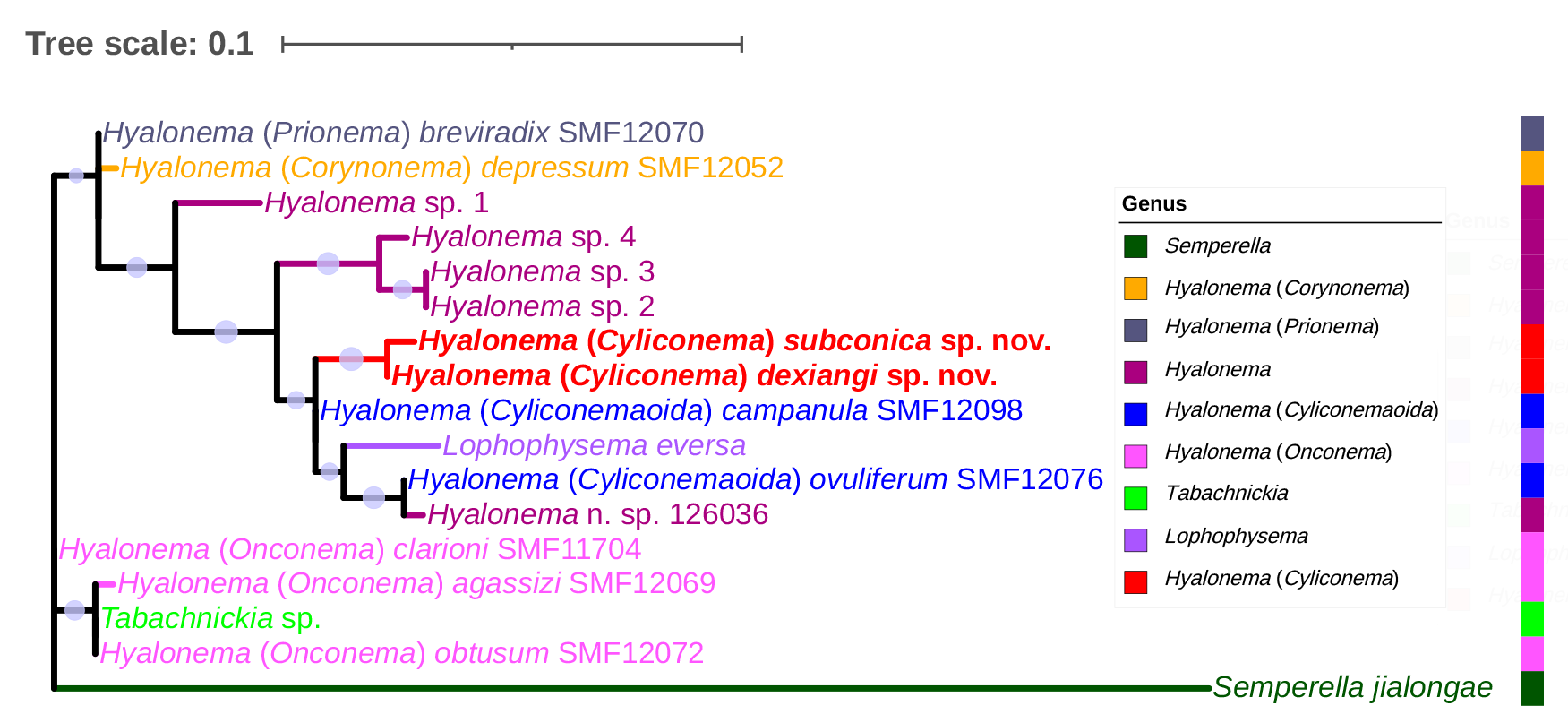

Supplement: Supplementary material 4 — Phylogenetic tree obtained by maximum-likelihood (ML) analysis based on 16S rDNA [file zookeys-1262-081_article-164821__-s004.tif]
